# An intelligent clustering method for devising the geochemical fingerprint of underground aquifers

**DOI:** 10.1016/j.heliyon.2021.e07017

**Published:** 2021-05-10

**Authors:** A. Di Roma, E. Lucena-Sánchez, G. Sciavicco, C. Vaccaro

**Keywords:** Geochemical fingerprinting, Aquifer fingerprinting, Intelligent clustering, Feature selection, Evolutionary algorithms

## Abstract

Geochemical fingerprinting is a rapidly expanding discipline in the earth and environmental sciences, anchored in the recognition that geological processes leave behind physical, chemical and sometimes also isotopic patterns in the samples. Furthermore, the geochemical fingerprinting of natural cycles (water, carbon, soil and biota fingerprinting) are influenced by the anthropogenic impact and by the climate change. So, their monitoring is a tool of resilience and adaptation. In recent years, computational statistics and artificial intelligence methods have started to be used to help the process of geochemical fingerprinting. In this paper we consider data from 57 wells located in the province of Ferrara (Italy), all belonging to the same geological group and separated into 4 different aquifers. The aquifer from which each well extracts its water is known only in 18 of the 57 cases, while in other 39 cases it can be only hypothesized based on geological considerations. We devise a novel technique for geochemical fingerprinting of groundwater by means of which we are able to identify the exact aquifer from which a sample is extracted with a sufficiently high accuracy. Then, we experimentally prove that out method is sensibly more accurate than typical statistical approaches, such as principal component analysis, for this particular problem.

## 1 Introduction

The increasing exploitation of water resources for human, industrial, and agricultural ends has brought in the last decades great attention toward the quality control of the groundwater [1], [2]. The complex reality of this sector has pushed the scientific community to take part in the study and the management of water resources, to improve the knowledge and to protect every realistic aspect of their management. The general intent is to deal with the problems originated by the variation of volumes and intensity of precipitation due to climate change, over-exploitation, salinization, anthropic pollution, degradation, and massive irrigation. An example of the need of a multidisciplinary approach is [3], but, in fact, many studies have demonstrated that a mindful protection of the existing water resources could contribute to the preservation of the availability of fresh water [4], [5], [6]. An hydro-geochemistry approach facilitates the understanding of the aquifer reborn, allowing to define the chemical composition of waters, and, through the application of specific models, to suspect and identify the presence of possible mixing between waters of different compositions. The quality of the water, and the geochemical fingerprint, of water bodies can be modified due to, for example, an interaction with a plume of polluted waters. A geochemical analysis allows one to identify the geochemical markers and to delimit the areas of diffusion of the plume and/or the intensity of the contamination, in order to quantify the impact and the risks.

Geologists usually develop a monitoring network, and, based on the sampling provided, they build a picture of the baseline conceptual hydrogeological model of the studied area, providing a prototype monitoring for continuous data acquisition. Then, *by hand*, sometimes with the help of basic statistical tools, they try to obtain the modeling of multi-aquifer flow in order to increase the knowledge of their hydrogeological characteristic, as well as to find the geochemical fingerprint that represents a specific aquifer level. This process is very expensive and entails an elevated risk of mistake due to potential loss of information, manual loading of data, and prolonged analysis time. In the recent literature, various statistical methods have been used to aid the traditional geochemical investigation to understand pollution sources, possible correlation among elements, and, in some cases, the nature of the contamination [7], [8], [9]. The recent work focused on protection of groundwater against pollution, deterioration, and for input pollution identification include applying geographical information systems and decision analysis [10], [11], logistic regression model learning [12], univariate and multivariate analysis [13], and multiple regression models [14]. More in general, machine learning is emerging as an effective, less complicated and less expensive [15], empirical approach for both regression and/or classification of nonlinear systems, ranging from few to thousands of variables, and they are ideal for addressing those problems where our theoretical knowledge is still incomplete but for which we do have a significant number of observations. In the past decades, it has proven useful for a very large number of applications, and among the techniques most commonly used we may mention artificial neural networks [16], [17], [18], [19], support vector machines [20], but also self-organizing map, decision trees, ensemble methods such as random forests, case-based reasoning, neuro-fuzzy networks, and evolutionary algorithms [21].

In this paper we considered 57 water wells located in the province of Ferrara, all belonging to the geological group *A* (the most superficial one), which, in turn, is separated into 4 different aquifers, named from *A*1 to *A*4 (see the stratigraphy made available by ENI-Agip, Regione Emilia Romagna and Eni-Agip deposit, 1998). The aquifer from which each well extracts its water is known only in 18 of the 57 cases, while in other 39 cases it can be only hypothesized based on geological considerations; the ultimate purpose of the present study it to devise an automatic, machine learning based method to identify the geochemical fingerprint of each aquifer, so that each unknown well can be assigned an aquifer, and the control network can be improved. This problem is associated to the well-known *clustering* problem, usually dealt with using classic statistical methods such as PCA [22], [23], [24], [25], [26]. In the typical setting, this problem is stated as follows: given samples of different aquifer, is there a geochemical fingerprint that allows one to identify the aquifers? The classic solution to this problem consists of applying a feature reduction and identification method, such as PCA, and then use the extracted features to design a fingerprint. In our case, however, the problem is different: we already know the geological structure, and we look for the best fingerprint that identifies each aquifer. Therefore, we cannot proceed as in classic way, which would imply disregarding the know geological group structure. For the purposes of this study, 910 samples were considered, 229 of which were extracted by a *single-filter* pump, and therefore can be used for this study. Each sample consists of 13 chemical-physical indicators. We search the geochemical fingerprint of each aquifer among combinations of these indicators and among combinations of *ratios* of affine elements and quantities. The number of possible combinations is exponential in the number of variables, giving rise to a feature selection problem combined with a clustering problem, which we express as an optimization problem and solve using an evolutionary algorithm. The result is the precise characterization of the geochemical fingerprint of each of the four aquifer, expressed in terms of *centroid*, that is, in terms of an ideal, hypothetical set of values for each aquifer of a selection of the indicators, that represents the aquifer itself. By using such a fingerprint, we were able to assign the correct aquifer to each of unknown wells, with a reasonable expected accuracy. Our approach differs from the classical clustering plus reduction one in several points: *(i)* in our case, the geological group is known, and we do not use clustering to identify the aquifer but, instead, their fingerprint; *(ii)* our reduction is dynamic: we search for the best subset towards fingerprint identification; *(iii)* we take into account possible non-linear contribution of each characteristic or ratio, improving the accuracy of the fingerprints.

This paper is organized as follows. In the next section, we give the necessary background on fingerprinting, feature selection, and clustering. In Section 3 we present our data and give a very simple exploratory analysis. In Section 4 we present the mathematical formulation of our technique: our results can be understood without the technical details of the method, which are however presented for completeness and reproducibility reasons. Then, in Section 5 we present our results, and we discuss them also via a simple comparison with those that can be obtained by existing approaches, before concluding.

## 2 Background

**Feature selection.** *Feature selection* is a machine learning technique for data preprocessing, defined as eliminating features from the data base that are irrelevant to the task to be performed [27]. In its original formulation and meaning, feature selection facilitates data understanding, reduces the storage requirements, and lowers the processing time, so that model learning becomes an easier process. Feature selection methods that do not incorporate dependencies between attributes are called *univariate* methods, and they consist in applying some criterion to each pair feature-response, and measuring the individual power of a given feature with respect to the response independently from the other features, so that each feature can be ranked accordingly. In *multivariate* methods, on the other hand, the assessment is performed for subsets of features rather than single features. There are several different approaches to feature selection in the literature. Among them, the most versatile ones are those that define the selection problem as an optimization problem. A *multi-objective optimization problem* (see, e.g. [28]) can be formally defined as the optimization problem of simultaneously minimizing (or maximizing) a set of *k* arbitrary functions:

(1) $$\left\{ \begin{array}{c} \min/\max\;f_{1}(\overset{¯}{x})\\ \min/\max\;f_{2}(\overset{¯}{x})\\ \ldots \\ \min/\max\;f_{k}(\overset{¯}{x}), \end{array}\right.$$

where $\overset{¯}{x}$ is a vector of decision variables. A multi-objective optimization problem can be *continuous*, in which we look for real values, or *combinatorial*, we look for objects from a countably (in)finite set, typically integers, permutations, or graphs. Maximization and minimization problems can be reduced to each other, so that it is sufficient to consider one type only. A set F of solutions for a multi-objective problem is *non dominated* (or *Pareto optimal*) if and only if for each $\overset{¯}{x}\in F$, there exists no $\overset{¯}{y}\in F$ such that *(i)* there exists *i* (1≤i≤k) that $f_{i}\left(\overset{¯}{y}\right)$ improves $f_{i}\left(\overset{¯}{x}\right)$, and *(ii)* for every *j*, (1≤j≤k, j≠i), $f_{j}\left(\overset{¯}{x}\right)$ does not improve $f_{i}\left(\overset{¯}{y}\right)$. In other words, a solution $\overset{¯}{x}$ *dominates* a solution $\overset{¯}{y}$ if and only if $\overset{¯}{x}$ is better than $\overset{¯}{y}$ in at least one objective, and it is not worse than $\overset{¯}{y}$ in the remaining objectives. We say that $\overset{¯}{x}$ is *non-dominated* if and only if there is not other solution that dominates it. The set of non dominated solutions from F is called *Pareto front*. Optimization problems can be approached in several ways; among them, *multi-objective evolutionary algorithms* are a popular choice (see, e.g. [29], [30], [31]).

Feature selection can be seen as a multi-objective optimization problem, in which the solution encodes the selected features, and the objective(s) are designed to evaluate the performances of some model-extraction algorithm; this may entail, for example, instantiating (1) as:

(2) $$\left\{ \begin{array}{c} \max\;Performance(\overset{¯}{x})\\ \min\;Cardinality(\overset{¯}{x}), \end{array}\right.$$

where $\overset{¯}{x}$ represents the chosen features; model (2) can be referred to as a *wrapper*. A typical concretization of a wrapper is feature selection for classification algorithms, whose performances are influenced by many factors, among which are the selected features. Evolutionary algorithms for feature selection have been reviewed [29], and a very recent survey of multi-objective algorithms for data mining in general can be found in [31]. An early evolutionary approach that includes the use of a multi-objective optimization algorithm for feature selection has been presented in [32], while a formulation of feature selection as a multi-objective optimization problem can be found in [30]. In [33] the authors proposed a wrapper-based approach that takes the error rate of the classifier as a whole and by-class, as well as the size of the subset, using multi-objective evolutionary computation, while the one proposed in [34] optimizes both the accuracy and the size of a decision tree. Another wrapper-based solutions were proposed in [35], [36], applied to the problem of cancer diagnosis are compared, and in [37], applied to automatic pattern classification. Other recent examples of multi-objective feature selection systems include [29], [38], [39].

**Centroid-based cluster analysis and** *KNN***.** *Cluster analysis* or *clustering* is the task of grouping a set of objects so that those in the same group, or *cluster* are more similar to each other than to those in other groups. The literature on cluster analysis is very wide, and includes *hierarchical* clustering, *centroid-based* models, *distribution-based* models, *density* models, among many others. Centroid-based models are of particular interest for us, because they are especially useful for numerical, many dimensional objects such as groundwater samples. The concept of centroid is essential in the most well-known centroid-based clustering algorithm, that is, *k-means* [40]: given a group of objects and a notion of distance, its *centroid* is the set of values that describes an object *C* (which may or may not be a concrete object of the group) such that the geometric mean of the distances between *C* and every other element of the group is minimal. In the *k*-means algorithm the groups (and even their number) is not known beforehand (this type of cluster analysis is called *exploratory*), and the algorithm is based on an initial random guessing of the centroid that eventually converges to a local optimum. *KNN* [41] is a distance-based *classification* algorithm, whose main idea is that close-by objects can be classified in a similar way. In this paper we use both ideas of centroid and distance-based classification in order to systematically extract geochemical fingerprints.

**Geochemical fingerprinting.** Geochemical fingerprinting is a rapidly expanding discipline in the earth and environmental sciences. It is anchored in the recognition that geological processes leave behind physical, chemical and sometimes also isotopic patterns in the samples. Many of these patterns, informally referred to as *geochemical fingerprints*, may differ only in fine details from each other. For this reason, approaching fingerprinting requires highly precise and accurate data analysis [42]. Applications of geochemical fingerprinting range on a wide set of contexts, from studies on ancient artifacts such as glass or ceramics [43], to mineral identification and discovery of Jurassic-age kimberlite [44], to dust transport monitoring [45], to groundwater resources identification and study [46]. Groundwater resources analysis has been the focus of studies aimed to fingerprinting for different purposes. In [47], for example, the authors use fingerprinting to evaluate the occurrence of microorganic elements and help understanding the sources and the processes which may be controlling the transport and fate of emerging contaminants in the floodplain of the River Thames to the northwest of Oxford and in the River Lambourn, in South-East England. In [48], top and subsoil groundwater were sampled around a station in Tomiño, in North-East Spain, and analyzed to identify and quantify volatile fuel organic compounds as well as diesel range organic elements. Also, in [49], discriminant analysis was used to identify the most probable source of chloride salinity in groundwater samples based on their geochemical fingerprints. Finally, geochemical fingerprinting proved itself relevant for the study of the quality of food and beverages, especially wine, as shown in [50].

Because of the statistical nature of geochemical fingerprints, statistical methods are suitable for their identification. In the most recent literature, statistical methods are being progressively integrated and paired with machine learning and artificial intelligence based technology. In this paper, we develop a novel method for groundwater fingerprint identification, based on feature selection, solved as an optimization problem, and implemented via a evolutionary algorithm.

## 3 Data and hydrogeological assessment

**Figure 1:**
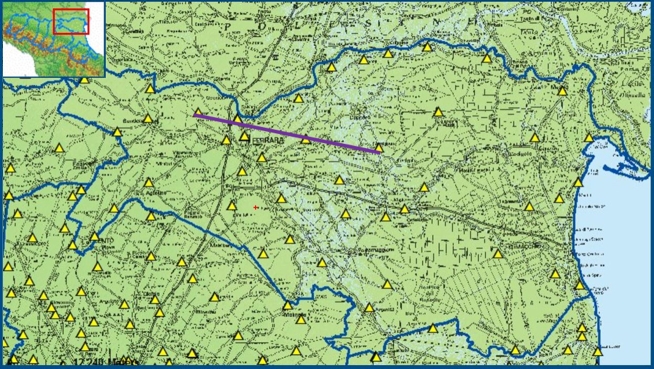
The area under study: aerial view.

The waters exploited for drinking purposes in northern Italy aquifers are contained in the Pliocene-Quaternary continental and marine Po deposit. This very important and valuable aquifer reservoir was the subject of extensive research over the past 20 years. Numerous studies have investigated the stratigraphic characteristics of the Po basin [51], [52]. The aquifers of the Emilia Romagna plain, in which the Po basin is partly inserted, consist mainly of alluvial deposits in the most superficial part of the plain, for a thickness of about 400-500 m, and, in minimal part, from marginal marine deposits. An areal view of the area of interest, located in the province of Ferrara (Emilia Romagna, Italy), can be seen in Fig. 1. With the purpose of characterizing the chemical state of the underground waters in this region, we have used data from the *regional waters monitoring program*, which are publicly available as per Italian Law 30/09. In order to be able to use all historical data from this program, we have verified, for each monitoring station, the structural characteristics, the depth, and the position of each filter. Whenever these details were not available, or the monitoring station presented more than one filter, it has been excluded from the study.

**Figure 2:**
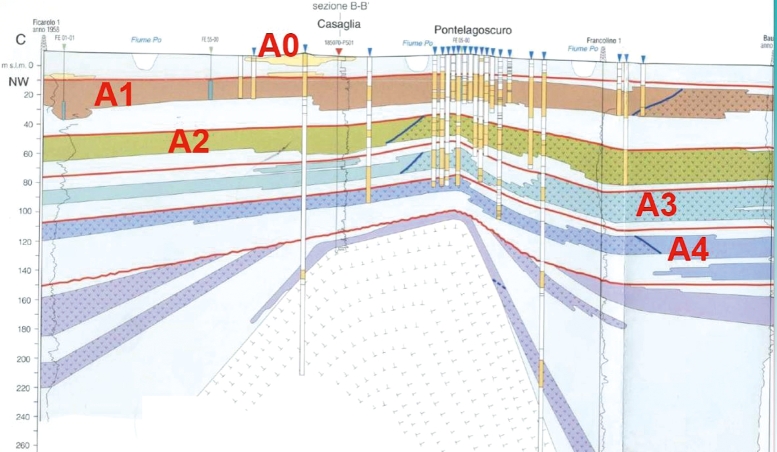
The area under study: cross-sectional view.

On the basis of the stratigraphy made available by ENI-Agip (Regione Emilia Romagna and Eni-Agip deposit, 1998) for hydrocarbons investigations, three aquifers groups were identified and referred to as *A*, *B* and *C*. The first two groups are located in the Quaternary deposits, while the aquifer system *C* belongs to Quaternary marine delta deposits. The data used for this study consist of 910 samples extracted from 57 wells located in the province of Ferrara, all belonging to the geological group *A* (the most superficial one), from 2010 to 2017. The hydro-stratigraphic units of interest, shown in Fig. 2, and named from *A*1 to *A*4 from the most to the least superficial one, are formed from one or more depositional sequences characterized by cyclic alternations of fine deposits (at the base) and coarser ones (the roof). Within each sequence, there are deposits composed by different lithologies, corresponding to various systems and depositional environments. At the base of each sequence is a very constant level to low permeability that acts as acquiclude, identified between the different units [53].

**Figure 3:**
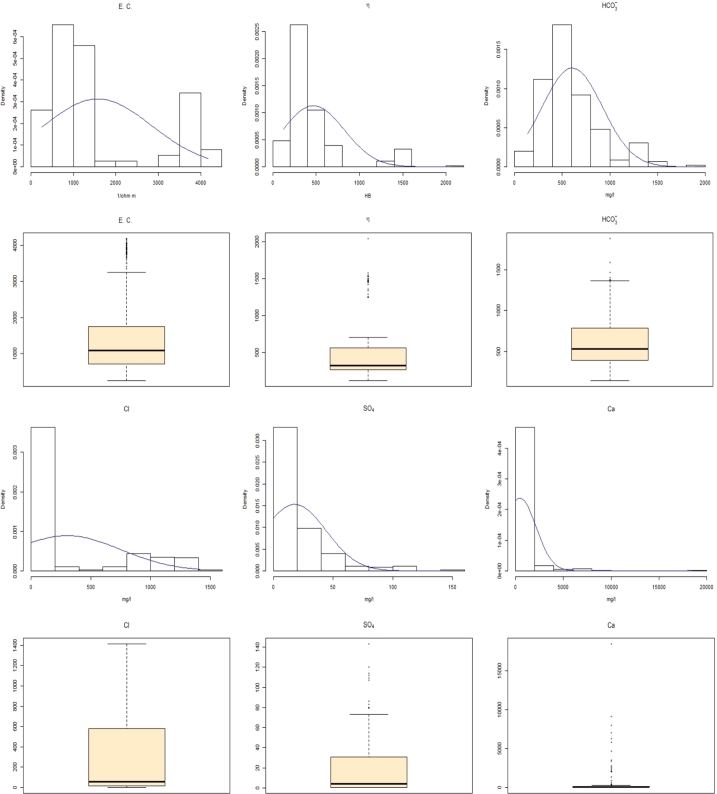
Distribution and outliers detection analysis: *E.C*., *η*, $HCO_{3}^{-}$, *Cl*, *SO*_4_, *Ca*.

**Figure 4:**
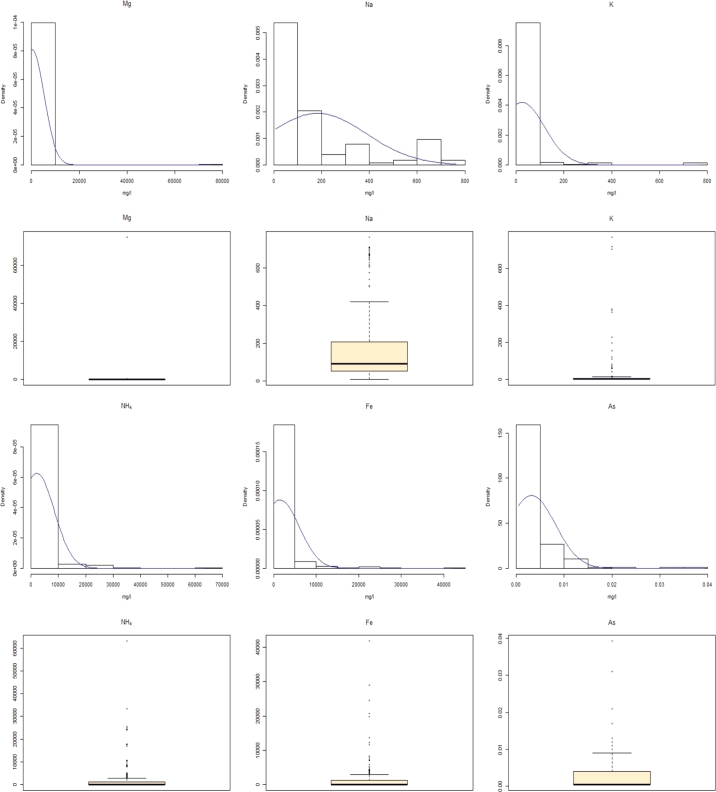
Distribution and outliers detection analysis: *Mg*, *Na*, *K*, *NH*_4_, *Fe*, *As*.

**Figure 5:**
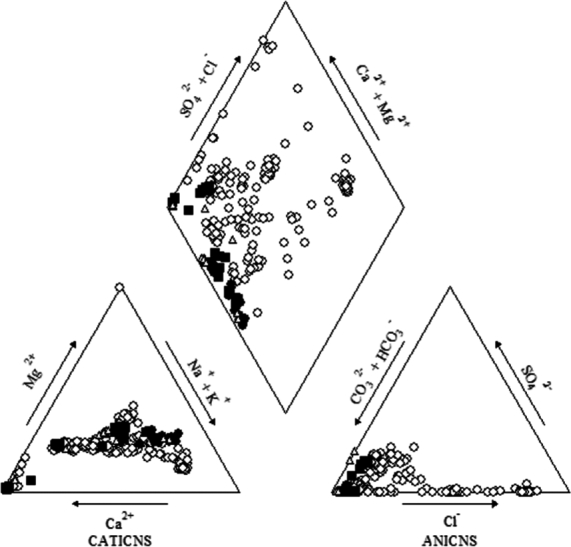
A Piper's diagram of the samples.

The exact aquifer from which each well extracts its water is known only in 18 of the 57 cases, while in other 39 cases it can be only hypothesized based on geological and stratigraphic considerations. Out of the total samples, we selected those which were extracted using single-filter pumps (that is, that give the guarantee that the groundwater comes from one aquifer only) and of which the precise aquifer was known, reducing our data set to 229 samples. Each sample contains 13 chemical-physical indicators: *η* (hardness), *T*, E.C., $Na^{+}$, $K^{+}$, $Ca^{2+}$, $Mg^{2+}$, $Cl^{-}$, $SO_{4}^{2-}$, $HCO_{3}^{-}$, $NH_{4}^{+}$, *Fe*, *As*. Data were already pre-processed, so no null values or low-variance columns have been found. Some relevant statistical measures of the different chemical elements are shown in Table 1: the (non-standardized) *mean*, the *p-value* associated with a Shapiro normality test (in which the null hypothesis is that the population is normally distributed), the *kurtosis*, and the *skewness* of each distribution. As it can be easily observed, none of the variables are normal (their *p*-values are well below 0.05), and they present a very high levels of kurtosis and skewness, being $Mg^{2+}$ and $Ca^{2+}$ the most evident examples. We show in Fig. 3 and Fig. 4 the graphical representation of the statistical behavior of each of the variables (except for the temperature, which presented the closest-to-normal behavior and it is therefore less informative). As it can be seen, the parameters show a very erratic behavior, with the presence of a relevant percentage of outliers [54]. The fact that most variables do not show a normal behavior can be considered as an argument against classical statistical methods for fingerprint extraction. The correlation between elements can be seen in Table 2; the most evident ones are electrical conductivity with $Cl^{-}$, and hardness with electrical conductivity and $HCO_{3}^{-}$. A Piper's diagram of the samples, that helps us understanding the hydrochemical facies of the geological group is shown in Fig. 5. As it can be seen, this geological group is characterized by a water mainly of a magnesium bicarbonate type, with no dominant cations facie, and a clear bicarbonate anion facie.

**Table 1:** Some basic statistical measures of our variables.

*feature*	*min*	*max*	*mean*	*p-value*	*kurtosis*	*skewness*
*η*	122.00	2038.00	469.50	7.01⁎10^−20^	7.18	2.15
*T*	11.20	20.00	15.81	1.16⁎10^−18^	5.37	0.55
*E.C*.	252.00	4175.00	1574.00	1.36⁎10^−18^	2.45	1.08
$HCO_{3}^{-}$	140.00	1879.00	606.50	3.54⁎10^−11^	4.44	1.18
*Cl*^−^	0.50	1413.00	56.00	2.43⁎10^−21^	2.81	1.25
$SO_{4}^{2-}$	0.50	143.00	18.05	1.61⁎10^−19^	7.59	2.03
*Ca*^2+^	26.00	18400.00	462.70	1.91⁎10^−29^	64.02	7.03
*Mg*^2+^	9.00	74740.00	381.33	2.89⁎10^−32^	226.95	15.03
*Na*^+^	8.00	763.30	183.52	1.79⁎10^−18^	4.02	1.51
*K*^+^	0.89	768.50	24.39	1.27⁎10^−29^	42.85	6.13
$NH_{4}^{+}$	0.00	63062.00	2135.61	2.01⁎10^−27^	43.06	5.51
*Fe*	0.00	41824.00	1501.52	1.40⁎10^−27^	39.20	5.47
*As*	0.01	0.04	3.23⁎10^−3^	1.38⁎10^−22^	18.84	3.23

**Table 2:** Correlation matrix.

	*T*	E.C*.*	*η*	$HCO_{3}^{-}$	$Cl^{-}$	$SO_{4}^{2-}$	$Ca^{2+}$	$Mg^{2+}$	$Na^{+}$	$K^{+}$	$NH_{4}^{+}$	*Fe*	*As*
*T*	1.000												
*E.C*.	0.050	1.000											
*η*	0.134	0.760	1.000										
$HCO_{3}^{-}$	0.069	0.479	0.786	1.000									
*Cl*^−^	0.026	0.956	0.625	0.249	1.000								
$SO_{4}^{2-}$	0.208	-0.329	-0.230	-0.344	-0.294	1.000							
*Ca*^2+^	0.002	0.189	0.141	0.109	0.159	-0.082	1.000						
*Mg*^2+^	0.038	0.102	0.024	-0.019	0.132	-0.038	-0.010	1.000					
*Na*^+^	-0.031	0.842	0.490	0.265	0.866	-0.364	-0.093	0.131	1.000				
*K*^+^	0.012	0.248	0.141	0.049	0.248	-0.061	0.767	-0.009	-0.093	1.000			
$NH_{4}^{+}$	-0.005	0.366	0.338	0.345	0.305	-0.213	-0.096	-0.030	0.332	-0.096	1.000		
*Fe*	0.053	0.357	0.462	0.472	0.269	-0.141	-0.054	0.007	0.223	-0.062	0.112	1.000	
*As*	-0.003	-0.206	-0.191	-0.073	-0.208	0.029	-0.051	-0.018	-0.154	-0.047	0.051	-0.004	1.000

As much as the temporal and spatial variability of our data are concerned, the following considerations are in order. On the one side, the observation period is less than 7 years from the first to the last sample. On the other side, the maximum distance between two extraction points is less than 22 km across. Since our approach is innovative, we first decided to ignore the differences that may emerge because of the temporal and the spatial variability. However, thanks to the very nature of the approach, both temporal and spatial variability can be taken into account with a minimal generalization of the method itself. At the end of Section 4, we briefly discuss such generalization.

## 4 Method

**Problem formulation.** Each instance in our data set can be seen as a vector in $R^{d}$; for example, in our case, we have that in the original data set d=13 because we consider the chemical-physical parameter as they appear in the samples. To evaluate the distance between two instances $I=(a_{1},\ldots ,a_{d})$ and $J=(a_{1}^{\prime },\ldots ,a_{d}^{\prime })$, we use the well-known notion of *Euclidean distance*, as in (3), below:

(3) $$dist(I,J)=\sqrt{\Sigma_{i=1}^{d}(|a_{i}-a_{i}^{\prime }{|}^{2})}$$

In this way we can compute the distance between any two samples of groundwater. Such a value is strongly influenced by the parameters (the specific subset of the *d* dimensions) that are taken into consideration. If we choose to represent the instances with a specific subset of parameters, instead of using all of them, the relative distances among different pairs of instances can vary very much. Our data set naturally entails a supervised classification problem, expressed as a matrix, as in (4):

(4) $$D=\left[\begin{array}{cccc} a_{11} & \ldots & a_{1d} & A1\\ \ldots & \ldots & \ldots \\ a_{m_{1}1} & \ldots & a_{m_{1}d} & A1\\ a_{(m_{1}+1)1} & \ldots & a_{(m_{1}+1)d} & A2\\ \ldots & \ldots & \ldots \\ a_{m_{2}1} & \ldots & a_{m_{2}d} & A2\\ a_{(m_{2}+1)1} & \ldots & a_{(m_{2}+1)d} & A3\\ \ldots & \ldots & \ldots \\ a_{m_{3}1} & \ldots & a_{m_{1}d} & A3\\ a_{(m_{3}+1)1} & \ldots & a_{(m_{3}+1)d} & A4\\ \ldots & \ldots & \ldots \\ a_{m_{4}1} & \ldots & a_{m_{4}d} & A4 \end{array}\right]$$

Consequently, the fingerprint extraction problem can be seen as a feature selection problem, that is, the problem of establishing the *best* subset of chemical-physical parameter. However, unlike the classical feature selection problem, selecting the correct classification algorithm (i.e., the correct inference model) is not immediate. We choose to model the fingerprint of an aquifer as the set of values that best represent an (ideal) sample of groundwater from that aquifer, that is, its centroid. Thus, we have a *feature selection for centroid identification* problem, as it is a clustering problem in which the clusters are already set.

**Multi-objective optimization problem formulation.** Let $\overset{¯}{x}=(x_{1},\ldots$, $x_{d})$ a vector of solution variables, each taking values in the domain {0,1}; as in a classical feature selection problem, each 1 means that the corresponding feature is selected, while 0 means that it is discarded; we denote by $C_{j}(\overset{¯}{x})$ the centroid of the *j*-th aquifer (1≤j≤4, in our case) computed using precisely the attributes that correspond to $\overset{¯}{x}$. In order to adapt (2) to our problem, we need to define how we evaluate the performances of the solution, which, in our case, means defining what classification problem we want to solve. To this end, indicating by A(I) the (true) aquifer to which the instance *I* correspond, we compute the *simple accuracy* of $\overset{¯}{x}$ over *D* as follows:

(5) $$SimpleAcc(\overset{¯}{x})=\Sigma_{I\in D}\left\{ \begin{array}{cc} 1\; & \text{if }A(I)=argmin_{A_{j}}\{d(I,C_{j})\}\\ 0\; & \text{otherwise} \end{array}\right.$$

Because of the particular nature of our problem, we can modify (5) to take into account that, although it is hypothesized the existence of impermeable layers between aquifers, infiltrations may occur. Therefore, a misclassification can be graded as less severe, in some sense, if the expected aquifer confines with the true one. The *adjusted accuracy* takes this aspect into account:

(6) $$AdjustedAcc(\overset{¯}{x})=\Sigma_{I\in D}\left\{ \begin{array}{cc} 1\; & \text{if }A(I)=argmin_{A_{j}}\{d(I,C_{j})\}\\ \frac{1}{2}\; & \text{if }A(I)=argmin_{A_{j}}\{d(I,C_{j})\}\pm 1\\ 0\; & \text{otherwise} \end{array}\right.$$

The two variants of the accuracy, namely (5) and (6) of a fingerprint selection can be used to reformulate our problem as an optimization problem, as they can both be seen as suitable performance indicators. Minimizing the cardinality of the selected features is also correct in fingerprinting selection, as smaller fingerprints are more interpretable. In order to take into account the fact that some geochemical processes are not necessarily linear, we can slightly complicate our formulation by introducing a third objective. As a matter of fact, we can expand the domain of each solution variable $x_{i}$ to take value in N, instead of {0,1}. While we still interpret 0 as discarding the corresponding parameter, we now interpret a positive value as the power to which the corresponding parameter is raised; we simulate, in this way, a sort of dynamic normalization of our data. In each execution, then, a vector of solutions variables $\overset{¯}{x}$ entails a transformation of the original data set (4) into:

(7) $$D=\left[\begin{array}{cccc} {a_{11}}^{x_{1}} & \ldots & {a_{1d}}^{x_{d}} & A1\\ \ldots & \ldots & \ldots \\ {a_{m_{1}1}}^{x_{1}} & \ldots & {a_{m_{1}d}}^{x_{d}} & A1\\ a_{11} & \ldots & a_{1d} & A2\\ \ldots & \ldots & \ldots \\ {a_{m_{2}1}}^{x_{1}} & \ldots & {a_{m_{2}d}}^{x_{d}} & A2\\ a_{11} & \ldots & {a_{1d}}^{x_{d}} & A3\\ \ldots & \ldots & \ldots \\ {a_{m_{3}1}}^{x_{1}} & \ldots & {a_{m_{1}d}}^{x_{d}} & A3\\ {a_{11}}^{x_{1}} & \ldots & {a_{1d}}^{x_{d}} & A4\\ \ldots & \ldots & \ldots \\ {a_{m_{4}1}}^{x_{1}} & \ldots & {a_{m_{2}d}}^{x_{d}} & A4 \end{array}\right]$$

where, for simplicity of notation, we have not shown the case of discarded attributes. There are two natural ways to optimize the complexity of the resulting fingerprint in terms of non-linear behavior, as in (7), that is, by minimizing the sum of all exponents:

(8) $$SumExp(\overset{¯}{x})=\Sigma_{i=1}^{d}{\overset{¯}{x}}_{i}$$

or the maximum exponent:

(9) $$MaxExp(\overset{¯}{x})=\max_{i=1}^{d}{\overset{¯}{x}}_{i}.$$

The objective functions (8) and (9) can be combined to obtain four variants of (2):

(10) $$\left\{ \begin{array}{c} \max\;SimpleAcc(\overset{¯}{x})\\ \min\;SumExp(\overset{¯}{x})\\ \min\;Cardinality(\overset{¯}{x}) \end{array}\right.$$

(11) $$\left\{ \begin{array}{c} \max\;AdjustedAcc(\overset{¯}{x})\\ \min\;SumExp(\overset{¯}{x})\\ \min\;Cardinality(\overset{¯}{x}) \end{array}\right.$$

(12) $$\left\{ \begin{array}{c} \max\;SimpleAcc(\overset{¯}{x})\\ \min\;MaxExp(\overset{¯}{x})\\ \min\;Cardinality(\overset{¯}{x}) \end{array}\right.$$

(13) $$\left\{ \begin{array}{c} \max\;AdjustedAcc(\overset{¯}{x})\\ \min\;MaxExp(\overset{¯}{x})\\ \min\;Cardinality(\overset{¯}{x}) \end{array}\right.$$

Models (10), (11), (12), and (13) will be tested and compared to each other in order to establish the best schema.

**Temporal and spatial generalization.** Our method can be seen as a *propositional* learning technique, in the sense that it is adimensional. In other words, possible temporal and spatial variations among values are ignored, and fingerprints are extracted by implicitly averaging the values over the whole period and the whole area under study. This may be acceptable in some cases (such as our one, for example: the accuracy that our fingerprints show proves that our approximation is acceptable). However, our approach is easily generalizable, to obtain a *more-than-propositional* method. Spatial variations of data are simply taken into account by, first, partitioning data into smaller areas, then solve the fingerprint problem per each area as above explained, and, finally, studying ow fingerprint are influenced by the physical positions of the wells. Instead, to take into account the temporal variability, the generalization would be as follows. First, every single extraction point would give rise not to different samples but a single multivariate temporal series, where each variable would be, as in the static case, a geochemical characteristic. Then, according to our schema, variables and non-linear contributions can be chosen within the optimization cycle, without disrupting the operations flow of the static case. Finally, an optimization problem can be defined in which the accuracy of the clustering algorithm is computed using some well-known notion of *distance* between time series [55], [56]. Observe that, in both cases, the structure of aquifer fingerprint would have the same aspect as in the static case. Lastly, it should be noticed that the more dimensions one wants to include in the model, the higher is the need in terms of number of samples.

**Limitations.** This approach can be used in a variety of situations, and should be considered as an aid to more classic fingerprint extraction methods. Its frequency-based nature frees it from pure statistical considerations (e.g., we do not assume normality of our variables), but, because of this, it needs a higher amount of samples than classic statistical approaches. Moreover, as it happens in our case, data are seldom balanced between classes; unbalanced data may lead to incorrect results, and re-balancing procedures have the ultimate effect of reducing the number of usable samples for training. Moreover, while temporal and spatial generalizations are possible, they do require a careful implementation and design.

## 5 Implementation, results and test

**Implementation and setting.** *Multi-objective evolutionary algorithms* are known to be particularly suitable to perform multi-objective optimization, as they search for multiple optimal solutions in parallel. In this experiment we have chosen the well-known NSGA-II (Non-dominated Sorted Genetic Algorithm) [57] algorithm, which is available as open-source from the suite *jMetal* [58]. NGSA-II is an elitist Pareto-based multi-objective evolutionary algorithm that employs a strategy with a binary tournament selection and a rank-crowding better function, where the rank of an individual in a population is the non-domination level of the individual in the whole population. We used the standard parameters in each experiment, and implemented elementary variants of mutation and crossover for them to be specific to our solution format. To cope with the intrinsic unbalancing of our data (over 70% of the samples belong to *A*1), we operated a re-sampling, to obtain a training set with 10 samples per each aquifer ($D_{training}$), and left every other sample for test ($D_{test}$). The test was performed by applying the accuracy function(s) to $D_{test}$ using the centroid and the selected attributes extracted from the chosen solution. For each of the four different multi-objective optimization models we have executed 10 runs, each with a different seed; the population side was 100 in each experiment, and we set each experiment for 100 generations each.

**Figure 6:**
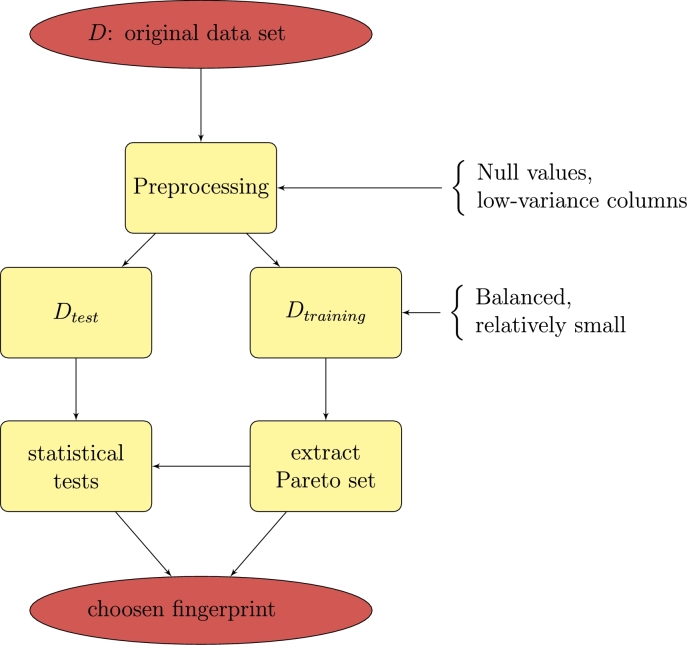
Simple schematics of the proposed methodology.

**Figure 7:**
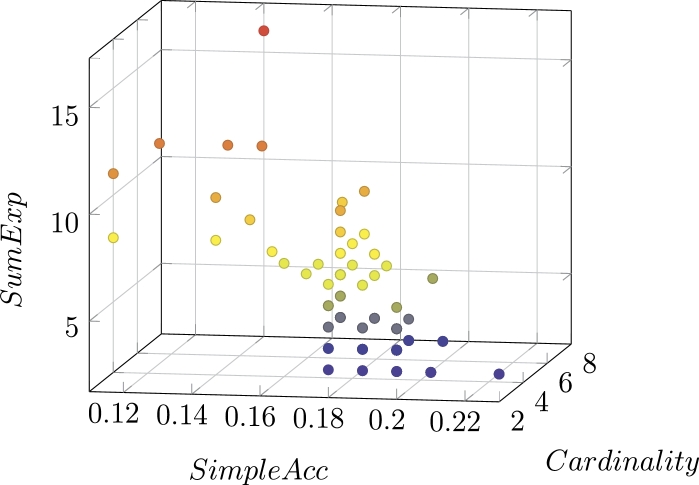
Example of Pareto front.

A multi-objective optimization problem gives rise to a *Pareto* front, that is, to a *last* population of (non-dominated) individuals from which one or more individuals can be selected via a decision-making process. An example of Pareto front in our case is shown in Fig. 7. As expected, it is 3-dimensional, as we have optimized three objectives; recall that, intuitively, each element of the front is a solution which cannot be improved by any of the objective without worsening at least one of the others (within the space of explored solutions of that particular run). The standard approach to decision making in a Pareto front is choosing one objective among all of them, and selecting the solution with the best value on that objective; in our case that would be the accuracy. Unfortunately, this strategy gives rise to fingerprints with too many characteristics, which would be not only too difficult to interpret, but also prone to overfitting, and hardly representable in a graphical way. Therefore, we selected the most accurate solution with strictly less than six elements. Our complete strategy, depicted in Fig. 6, consists of: preprocessing the original data set, dealing with null values (we have chosen to eliminate every record with at least one value) and low-variance columns (in our data set all columns present sufficient variance), separating it into training and test subsets (as explained before), performing the fingerprint extraction, selecting the best element(s) from the Pareto front(s), and returning it (them) for interpretation.

**Figure 8:**
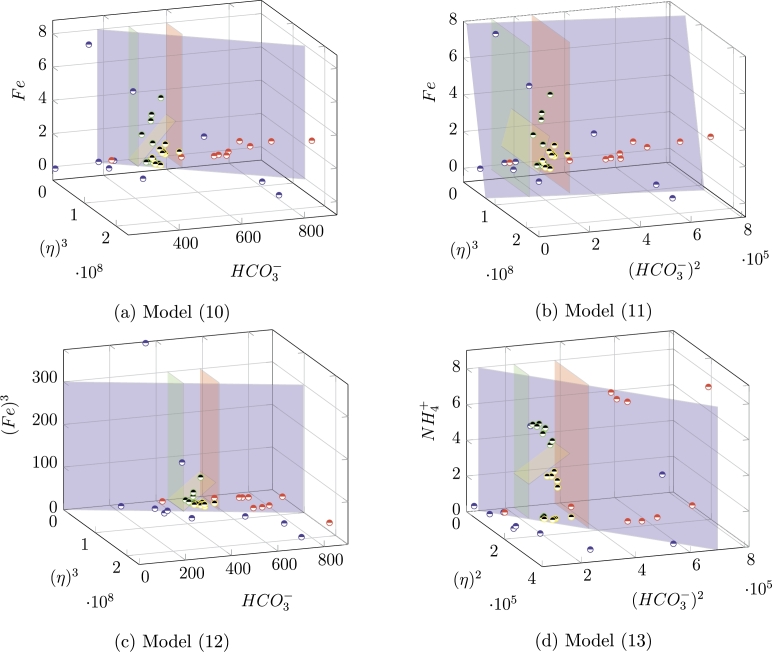
Graphical representation of the starred solutions from Table 3. Figure (*a*): $\eta^{3}\cdot 10^{8},HCO_{3}^{-},Fe$. Figure (*b*): $\eta^{3}\cdot 10^{8},HC{O_{3}^{-}}^{2}\cdot 10^{5},Fe$. Figure (*c*): $\eta^{3}\cdot 10^{8},HCO_{3}^{-},Fe^{3}$. Figure (*d*): $\eta^{2}\cdot 10^{5},HC{O_{3}^{-}}^{2}\cdot 10^{5},NH_{4}^{+}$.

**Characteristics-based fingerprinting.** For this set of experiments we used $D_{training}$ and $D_{test}$ without any further transformation. We obtained four sets of results, displayed in Table 3. The column *acc* indicates the obtained accuracy in test, and the remaining columns show how this accuracy becomes when analyzed by class, that is, how accurate is our model in identifying each of the four aquifers. As it can be observed, the general accuracy ranges from 0.54 to 0.66, which can be considered relatively high. By looking as the single class results, as it turns out, the aquifers *A*3 and *A*4 are identified with accuracies from 0.9 to 1, and aquifer *A*2 is correctly identified with a rate from 0.71 to 0.81. Aquifer *A*1 seems to be the most difficult one. For each model, we identified the *simplest most accurate* solution whose result can be displayed, looking at the highest accuracies within fingerprints with less than four columns, and with a preference for lower exponents - these are indicated by a ^⁎^ in Table 3. In Fig. 8, such solutions are displayed in a graphical way, where their distinguishing power becomes evident. These results can be explained as follows. The $Ca^{2+}$ and $HCO_{3}^{-}$ levels are controlled by the interaction between rock and water, and related to the dissolution of carbonate and to the degradation of organic matter [59]. Hydrogen carbonate, in particular, is the dissolved inorganic carbon in fresh waters, which derives from the dissolution of calcite and dolomite, and its levels, therefore, implicitly express the concentrations of calcium and magnesium derived from these two minerals. Moreover, calcium and magnesium, together, define the level of hardness (*η*) of the water. Finally, iron and manganese, among others, are widely found in soils and aquifers, and have similar geochemical behavior. The reducing conditions, residence time, well depth, and salinity are the key factors leading the dissolution and migration of *Fe* and *Mn* to groundwater [60]. This may explain our findings, that seem to indicate the $HCO_{3}^{-}$, *Fe*, and *η*, allow one to distinguish between the aquifers of the group under analysis.

**Table 3:** Results of the characteristics-based fingerprinting experiment. From top to bottom: model (10), (11), (12), and (13). Starred results are the best ones of each model.

			*recall*			
	*fingerprint*	*acc.*	*A*1	*A*2	*A*3	*A*4

*model*(10)	*η*, ${\left(HCO_{3}^{-}\right)}^{3}$, ${\left(NH_{4}^{+}\right)}^{2}$, (*Fe*)^2^	0.60	0.55	0.71	1.00	1.00
	*η*, ${\left(HCO_{3}^{-}\right)}^{3}$, ${\left(NH_{4}^{+}\right)}^{2}$, *Fe*	0.60	0.55	0.71	1.00	1.00
	(*η*)^3^, ${\left(HCO_{3}^{-}\right)}^{2}$, (*Fe*)^3^	0.60	0.55	0.71	0.90	1.00
	(*T*)^2^, *η*, ${\left(HCO_{3}^{-}\right)}^{3}$	0.55	0.49	0.81	0.90	0.75
	*η*, ${\left(HCO_{3}^{-}\right)}^{2}$, ${\left(Na^{+}\right)}^{2}$, $NH_{4}^{+}$	0.54	0.47	0.81	1.00	1.00
	(*η*)^3^, $HCO_{3}^{-}$, *Fe*	0.60	0.55	0.71	0.90	1.00
	(*η*)^3^, ${\left(HCO_{3}^{-}\right)}^{2}$, (*Fe*)^3^	0.60	0.55	0.71	0.90	1.00
	(*T*)^2^, *η*, $HCO_{3}^{-}$	0.55	0.49	0.81	0.90	0.75
	*η*, ${\left(HCO_{3}^{-}\right)}^{3}$, ${\left(Na^{+}\right)}^{3}$, (*Fe*)^3^	0.54	0.47	0.71	1.00	1.00
⁎	(*η*)^3^, $HCO_{3}^{-}$, *Fe*	0.60	0.55	0.71	0.90	1.00

*model*(11)	(*η*)^2^, ${\left(HCO_{3}^{-}\right)}^{3}$, ${\left(NH_{4}^{+}\right)}^{3}$, (*Fe*)^2^	0.66	0.55	0.71	1.00	1.00
	(*η*)^2^, ${\left(HCO_{3}^{-}\right)}^{3}$, (*Fe*)^3^, (*As*)^2^	0.66	0.55	0.71	0.90	1.00
	(*η*)^2^, $HCO_{3}^{-}$, ${\left(NH_{4}^{+}\right)}^{2}$	0.64	0.53	0.81	1.00	1.00
	(*η*)^2^, ${\left(HCO_{3}^{-}\right)}^{3}$, $NH_{4}^{+}$, (*Fe*)^2^	0.66	0.55	0.71	1.00	1.00
	*η*, ${\left(HCO_{3}^{-}\right)}^{2}$, ${\left(Na^{+}\right)}^{2}$, ${\left(NH_{4}^{+}\right)}^{3}$, (*As*)^2^	0.62	0.47	0.81	1.00	1.00
	(*η*)^2^, ${\left(HCO_{3}^{-}\right)}^{3}$, $NH_{4}^{+}$, *Fe*	0.66	0.55	0.71	1.00	1.00
	(*η*)^2^, ${\left(HCO_{3}^{-}\right)}^{3}$, ${\left(NH_{4}^{+}\right)}^{3}$, (*Fe*)^2^	0.66	0.55	0.71	1.00	1.00
	(*η*)^2^, ${\left(HCO_{3}^{-}\right)}^{3}$, $NH_{4}^{+}$, (*Fe*)^3^	0.66	0.55	0.71	1.00	1.00
⁎	(*η*)^3^, ${\left(HCO_{3}^{-}\right)}^{2}$, *Fe*	0.66	0.55	0.71	0.90	1.00
	*η*, ${\left(HCO_{3}^{-}\right)}^{2}$, ${\left(Na^{+}\right)}^{2}$, ${\left(NH_{4}^{+}\right)}^{2}$, (*Fe*)^3^	0.62	0.47	0.81	1.00	1.00

*model*(12)	(*η*)^2^,$HCO_{3}^{-}$, ${\left(NH_{4}^{+}\right)}^{3}$	0.59	0.53	0.48	1.0	1.00
	(*η*)^3^, $HCO_{3}^{-}$, (*Fe*)^3^	0.60	0.55	0.43	0.90	1.00
	(*η*)^3^, ${\left(HCO_{3}^{-}\right)}^{3}$, (*Fe*)^3^	0.60	0.55	0.43	0.90	1.00
	(*η*)^2^, ${\left(HCO_{3}^{-}\right)}^{3}$, ${\left(NH_{4}^{+}\right)}^{2}$, *Fe*	0.60	0.55	0.48	0.90	1.00
	(*T*)^2^, ${\left(HCO_{3}^{-}\right)}^{2}$, $SO_{4}^{2-}$, $NH_{4}^{+}$	0.54	0.48	0.43	1.00	0.75
	(*η*)^3^, ${\left(HCO_{3}^{-}\right)}^{3}$, (*Fe*)^3^	0.60	0.55	0.43	0.90	1.00
	(*η*)^3^, ${\left(HCO_{3}^{-}\right)}^{3}$, (*Fe*)^3^	0.60	0.55	0.43	0.90	1.00
	(*T*)^2^, *η*, ${\left(HCO_{3}^{-}\right)}^{2}$	0.55	0.49	0.43	1.00	0.75
⁎	(*η*)^3^, $HCO_{3}^{-}$, (*Fe*)^3^	0.60	0.55	0.43	0.90	1.00
	(*T*)^2^, *η*, $HCO_{3}^{-}$	0.55	0.49	0.43	1.00	0.75

*model*(13)	(*η*)^2^, ${\left(HCO_{3}^{-}\right)}^{3}$, ${\left(NH_{4}^{+}\right)}^{3}$, (*Fe*)^2^	0.66	0.55	0.71	1.00	1.00
	*η*, $HCO_{3}^{-}$, $NH_{4}^{+}$, *Fe*	0.64	0.54	0.81	0.90	1.00
⁎	(*η*)^2^, ${\left(HCO_{3}^{-}\right)}^{2}$, $NH_{4}^{+}$	0.64	0.53	0.81	1.00	1.00
	(*η*)^2^, ${\left(HCO_{3}^{-}\right)}^{3}$, ${\left(NH_{4}^{+}\right)}^{2}$, $NH_{4}^{+}$, (*As*)^2^	0.66	0.55	0.71	1.00	1.00
	*η*, ${\left(HCO_{3}^{-}\right)}^{2}$, ${\left(Na^{+}\right)}^{2}$, ${\left(NH_{4}^{+}\right)}^{3}$, (*As*)^2^	0.62	0.47	0.81	1.00	1.00
	*η*, ${\left(HCO_{3}^{-}\right)}^{2}$, ${\left(Na^{+}\right)}^{2}$, ${\left(NH_{4}^{+}\right)}^{3}$	0.62	0.47	0.81	1.00	1.00
	(*η*)^2^, ${\left(HCO_{3}^{-}\right)}^{2}$, ${\left(NH_{4}^{+}\right)}^{2}$	0.64	0.53	0.81	1.00	1.00
	*η*, ${\left(HCO_{3}^{-}\right)}^{2}$, *Na*^+^, ${\left(NH_{4}^{+}\right)}^{2}$, (*Fe*)^2^, (*As*)^3^	0.62	0.47	0.81	1.00	1.00
	(*η*)^2^, ${\left(HCO_{3}^{-}\right)}^{3}$, ${\left(NH_{4}^{+}\right)}^{2}$, (*Fe*)^3^	0.66	0.55	0.71	1.00	1.00
	*η*, ${\left(HCO_{3}^{-}\right)}^{2}$, ${\left(Na^{+}\right)}^{2}$, $NH_{4}^{+}$, (*Fe*)^2^	0.62	0.47	0.81	1.00	1.00

**Figure 9:**
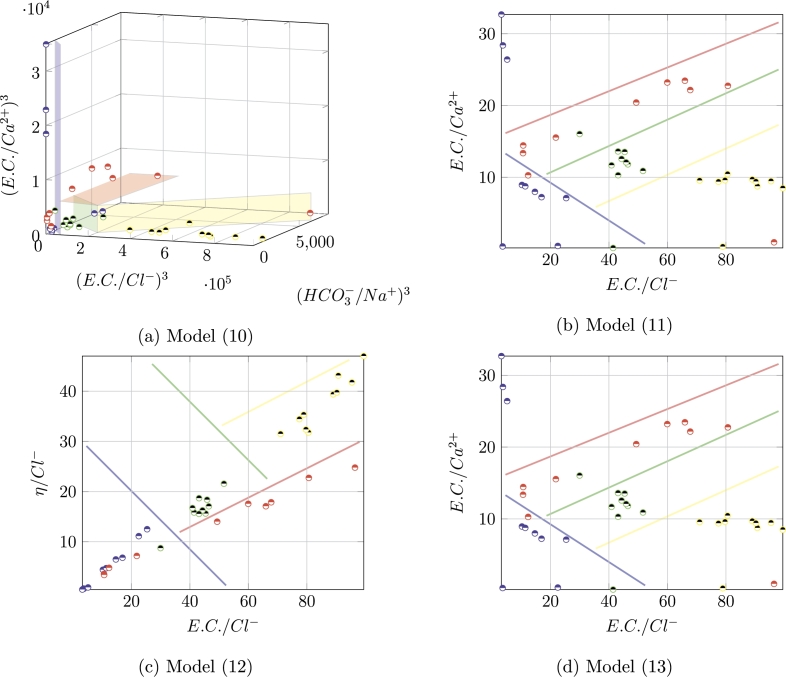
Graphical representation of the starred solutions from Table 4. Figure (*a*): ${\left(E.C./Ca^{2+}\right)}^{3}\cdot 10^{5},{\left(E.C./Cl^{-}\right)}^{3}\cdot 10^{4}$. Figure (*b*): (*E.C*./*Ca*^2+^),(*E.C*./*Cl*^−^). Figure (*c*): (*η*/*Cl*^−^),(*E.C*./*Cl*^−^). Figure (*d*): (*E.C*./*Ca*^2+^),(*E.C*./*Cl*^−^).

**Ratios-based fingerprinting.** The fingerprinting problem can be also approached by looking into subsets of *ratios* among characteristics, instead of subsets of characteristics. In other words, instead of looking for the geochemical fingerprint of each aquifer among combinations of the chemicals indicators, we search it among combinations of ratios of affine elements and quantities. This entails pre-processing the data set to compute such ratios, and then applying the same optimization models. The reason behind this approach lies in the fact that combinations of ratios of affine elements can sometimes better identify the geochemical signatures of an aquifer, as they tend to be preserved during the dilution contribution of meteoric waters of reborn. In Table 4 we show the ten executions with the four models. As it can be seen, some of the proposed solutions present very high accuracies; general accuracy now ranges from 0.79 to 0.91, and aquifer *A*1 is now correctly identified with accuracies from 0.75 to 0.91. For those fingerprints with three elements or less, their ability of discernment can be also shown graphically (see Fig. 9); thus, we show the simplest most accurate solution for each model in this case as well. As we can see, using ratios increases in a sensible way the accuracy of our characterization; the ratios that emerged as those with better discernment ability can be explained as follows. First, electrical conductivity has been used to characterize groundwater by several authors [61], [62], the presence of inorganic suspended solids such as chloride, nitrate, phosphate, and sulfate ions (ions that carry a negative charge), or aluminum, calcium, magnesium, iron, and sodium ions (ions that carry a positive charge) may affect electrical conductivity. Electrical conductivity values may reflect both the recharge dynamics and the possible excessive pumping of wells. Second, the concentration ratios between alkaline earth elements could be correlated to water-sediment interaction times, and the contribution of fossil water content trapped in deep sediments [63]. In conclusions, the fingerprints that emerge from our analysis seem to indicate that the different aquifers can be distinguished from these elements: freshwater/fossil water mixing and saltwater contamination.

**Table 4:** Results of the ratios-based fingerprinting experiment. From top to bottom: model (10), (11), (12), and (13). Starred results are the best ones of each model.

			*recall*			
	*fingerprint*	*acc.*	*A*1	*A*2	*A*3	*A*4

*model*(10)	*E.C*./*Cl*^−^, $HCO_{3}^{-}$/*Ca*^2+^	0.84	0.90	0.52	0.50	1.00
	${\left(E.C./Cl^{-}\right)}^{3}$, ${\left(\eta /Cl^{-}\right)}^{2}$, ${\left(Na^{+}/T\right)}^{2}$, ${\left(HCO_{3}^{-}/Ca^{2+}\right)}^{2}$	0.86	0.90	0.52	0.80	1.00
⁎	${\left(E.C./Cl^{-}\right)}^{3}$, ${\left(HCO_{3}^{-}/Na^{+}\right)}^{3}$, ${\left(E.C./Ca^{2+}\right)}^{3}$	0.85	0.90	0.48	0.80	1.00
	${\left(\eta /Cl^{-}\right)}^{3}$, $HCO_{3}^{-}$/*Ca*^2+^	0.79	0.81	0.62	0.90	1.00
	*E.C*./*Cl*^−^, *η*/*Cl*^−^, (*η*/*T*)^3^	0.85	0.91	0.43	0.80	1.00
	${\left(Cl^{-}/Na^{+}\right)}^{2}$, *E.C*./*Cl*^−^, *Na*^+^/*T*, ${\left(HCO_{3}^{-}/\eta \right)}^{2}$	0.83	0.90	0.43	0.50	1.00
	${\left(E.C./Cl^{-}\right)}^{3}$, ${\left(HCO_{3}^{-}/T\right)}^{2}$	0.79	0.79	0.67	0.90	1.00
	${\left(E.C./Cl^{-}\right)}^{2}$, ${\left(\eta /Cl^{-}\right)}^{2}$	0.85	0.90	0.48	0.80	1.00
	${\left(E.C./Cl^{-}\right)}^{2}$, *Ca*^2+^/*K*^+^, ${\left(E.C./Ca^{2+}\right)}^{2}$	0.75	0.82	0.24	0.70	1.00
	${\left(E.C./Cl^{-}\right)}^{3}$, ${\left(HCO_{3}^{-}/T\right)}^{3}$, (*η*/*T*)^3^	0.84	0.86	0.62	0.90	1.00

*model*(11)	*η*/*Cl*^−^, ${\left(\eta /Na^{+}\right)}^{3}$	0.90	0.82	0.43	0.80	1.00
	${\left(NH_{4}^{+}\right)}^{2}$, ${\left(E.C./Cl^{-}\right)}^{3}$, ${\left(\eta /Cl^{-}\right)}^{3}$	0.91	0.90	0.48	0.70	1.00
	${\left(\eta /Cl^{-}\right)}^{2}$, *E.C*./*Ca*^2+^	0.88	0.75	0.57	0.90	1.00
	${\left(E.C./Cl^{-}\right)}^{3}$, ${\left(\eta /Cl^{-}\right)}^{3}$, *Na*^+^/*T*	0.87	0.90	0.43	0.80	1.00
	${\left(E.C./Cl^{-}\right)}^{3}$, ${\left(\eta /Cl^{-}\right)}^{2}$,${\left(\eta /Na^{+}\right)}^{3}$	0.91	0.90	0.48	0.80	1.00
	${\left(E.C./Cl^{-}\right)}^{3}$, *Na*^+^/*T*, *η*/*T*	0.89	0.76	0.67	1.00	1.00
	${\left(E.C./Cl^{-}\right)}^{2}$, ${\left(HCO_{3}^{-}/\eta \right)}^{3}$, ${\left(HCO_{3}^{-}/T\right)}^{3}$	0.86	0.79	0.67	0.90	1.00
	*Cl*^−^/*Na*^+^, *E.C*./*Cl*^−^, *Na*^+^/*T*	0.91	0.90	0.43	0.50	1.00
	*E.C*./*Cl*^−^, *η*/*Cl*^−^, (*η*/*T*)^3^	0.90	0.91	0.43	0.80	1.00
⁎	*E.C*./*Cl*^−^, *E.C*./*Ca*^2+^	0.91	0.90	0.48	0.60	1.00

*model*(12)	${\left(Cl^{-}/Na^{+}\right)}^{2}$, ${\left(E.C./Cl^{-}\right)}^{2}$, ${\left(\eta /Cl^{-}\right)}^{2}$	0.85	0.90	0.48	0.80	1.00
⁎	*E.C*./*Cl*^−^, ${\left(\eta /Cl^{-}\right)}^{2}$	0.85	0.90	0.48	0.80	1.00
	$NH_{4}^{+}$, *E.C*./*Cl*^−^, *η*/*T*	0.83	0.90	0.38	0.50	1.00
	${\left(NH_{4}^{+}\right)}^{3}$, ${\left(\eta /Cl^{-}\right)}^{2}$, ${\left(HCO_{3}^{-}/Ca^{2+}\right)}^{2}$, *E.C*./*η*	0.81	0.83	0.67	0.80	1.00
	*T*, ${\left(\eta /Cl^{-}\right)}^{2}$, $HCO_{3}^{-}$/*Ca*^2+^	0.79	0.80	0.62	0.90	1.00
	*η*/*Cl*^−^, $HCO_{3}^{-}$/*Ca*^2+^	0.79	0.81	0.62	0.90	1.00
	*As*, *E.C*./*Cl*^−^, *E.C*./*Na*^+^	0.84	0.91	0.48	0.50	1.00
	${\left(E.C./Cl^{-}\right)}^{2}$, ${\left(Na^{+}/T\right)}^{2}$	0.83	0.90	0.43	0.50	1.00
	*η*/*Cl*^−^, *E.C*./*Ca*^2+^	0.75	0.75	0.57	0.90	1.00
	*η*/*Cl*^−^, $HCO_{3}^{-}$/*T*	0.66	0.60	0.81	1.00	1.00

*model*(13)	${\left(E.C./Cl^{-}\right)}^{2}$, ${\left(E.C./Ca^{2+}\right)}^{2}$	0.90	0.90	0.48	0.60	1.00
	*E.C*./*Cl*^−^, *η*/*Cl*^−^, $HCO_{3}^{-}$/*Na*^+^	0.91	0.90	0.48	0.80	1.00
	${\left(NH_{4}^{+}\right)}^{2}$, ${\left(\eta /Cl^{-}\right)}^{3}$, ${\left(HCO_{3}^{-}/Ca^{2+}\right)}^{3}$, ${\left(E.C./HCO_{3}^{-}\right)}^{3}$	0.88	0.82	0.67	0.80	1.00
	${\left(\eta /Cl^{-}\right)}^{3}$, ${\left(HCO_{3}^{-}/Ca^{2+}\right)}^{2}$, ${\left(T/K^{+}\right)}^{2}$	0.87	0.81	0.62	1.00	1.00
	*E.C*./*Cl*^−^, *η*/*Cl*^−^	0.91	0.90	0.48	0.80	1.00
	${\left(E.C./Cl^{-}\right)}^{3}$, ${\left(\eta /Cl^{-}\right)}^{2}$	0.91	0.90	0.48	0.80	1.00
	(*η*)^2^, ${\left(HCO_{3}^{-}\right)}^{3}$, *η*/*Cl*^−^, ${\left(HCO_{3}^{-}/Ca^{2+}\right)}^{3}$	0.89	0.88	0.57	0.90	1.00
	*η*/*Cl*^−^, ${\left(HCO_{3}^{-}/Ca^{2+}\right)}^{3}$, *η*/*T*	0.86	0.78	0.62	0.90	1.00
	*E.C*./*Cl*^−^, ${\left(\eta /Cl^{-}\right)}^{2}$, *η*/*T*	0.91	0.91	0.43	0.80	1.00
⁎	*E.C*./*Cl*^−^, *E.C*./*Ca*^2+^	0.90	0.90	0.48	0.60	1.00

**Choosing fingerprints.** After our analysis of the results, it seems clear that the following pair of ratios:

$$E.C./Cl^{-},E.C./Ca^{2+}$$

is able to distinguish with the most accuracy the four aquifers. By reading the linear boundaries between values, one obtains that each aquifer is characterized by the following intervals:

$$\begin{array}{ccc} A1:\; & \text{under}\; & E.C./Ca^{2+}=-0.272\cdot E.C./Cl^{-}+14.251\\ A2:\; & \text{between}\; & E.C./Ca^{2+}=0.16\cdot E.C./Cl^{-}+7.267\text{ and }\\ \; & \; & E.C./Ca^{2+}=0.1748\cdot E.C./Cl^{-}+15.475\\ A3:\; & \text{between}\; & E.C./Ca^{2+}=0.161\cdot E.C./Cl^{-}+0.049\text{ and}\\ \; & \; & E.C./Ca^{2+}=0.16\cdot E.C./Cl^{-}+7.267\\ A4:\; & \text{under}\; & E.C./Ca^{2+}=0.161\cdot E.C./Cl^{-}+0.049 \end{array}$$

**Figure 10:**
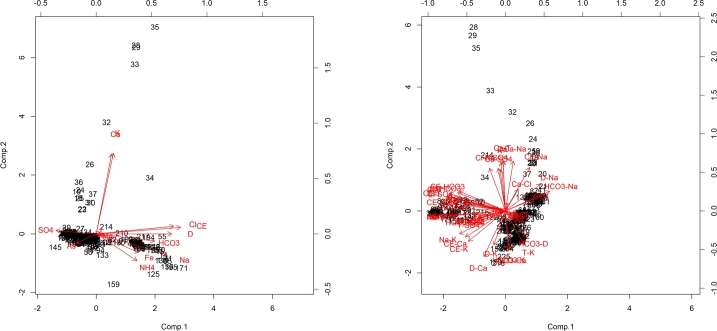
Graph of the first two principal components of the PCA over the original data set. Left-hand side: characteristics-based PCA. Right-hand side: ratios-based PCA.

**Comparison with classical statistical methods.** Principal component analysis followed by clustering is the most classical approach to problems similar to the one we have considered here [23], [24], [25], [26]. However, in the classical setting, the geological group from which samples are taken is now completely known, or must be confirmed. Clustering, that is, finding the number of clusters, their centroids, and associating every sample to its centroid, is a typical approach when aquifers must be identified or confirmed; principal component analysis is applied as a preliminary step to reduce the number of variables to be taken into account. So, in the classical setting, the number of clusters must be guessed, as well as their centroids. To compare our results with those that can be obtained with existing approaches, then, we apply principal components analysis only: since our geological group is known, their centroids are also known, and only fingerprints remain to be discovered. *Principal component analysis* (or *PCA* [64], [65]) is a technique for reducing the dimensionality of a data set, increasing interpretability but at the same time minimizing information loss. It does so by creating new uncorrelated variables that successively maximize variance. Finding such new variables, the *principal components*, reduces to solving an eigenvalue/eigenvector problem, and the new variables are defined by the data set. PCA has been successfully used in pure fingerprinting in the recent literature [66], [67]. Although it does not explicitly assume Gaussian distribution of the variables, PCA is only concerned with variance; non-normal distributions (such as those shown in our data) have higher order statistic beyond variance which are not taken into account in this analysis, leading to the conclusion that applying such classical tool may return possibly unreliable results. Moreover, as a purely statistical approach, it does not always offer the elasticity required to test the performance of a solution; in other words, there are no systematic methods to reduce the elements in a principal component, or taking into account non-linear underlying processes. We have applied the algorithm for PCA available in the well-known learning suite R [68] to the entire data set *D* of characteristics; Fig. 10 (left-hand side) represents the loads and standardized scores of the first two principal components, resulting into a two-dimensional projection of the initial axes of the variables and the (standardized) scores of the individuals in the data. If we choose as fingerprint the union of the first two component, which explain the 93% of the variance in our the data, we obtain the following signature:

$$E.C.,\eta ,Cl^{-},Ca^{2+},Mg^{2+},Na^{+},$$

which has a simple accuracy of 0.45. As it can be observed, our method gives us a much better result (from 0.54 to 0.66). If we focus on the recall of the fingerprint produced by the PCA, we find that it is 0.38, 0.76, 0.00, and 0.75, respectively, for A1,A2,A3 and *A*4, which are generally worse than the values obtained by our fingerprints. For completeness of exposition, it should also be pointed out that the PCA was computed on the entire data set; in machine learning terms, this means that the obtained value is to be considered *full training* mode, while the values in Table 3 are in *training+test* mode. Usually, full training results are better in terms of absolute accuracy, but less generalizable (that is, they tend to overfit). In other words, our fingerprints are more accurate, and more reliable solutions. PCA applied to the data set with the ratios resulted as in Fig. 10 (right-hand side); applying similar criteria as in the previous case gives us the following fingerprint:

$$E.C.,Cl^{-}/SO_{4}^{2-},HCO_{3}^{-}/SO_{4}^{2-},E.C./SO_{4}^{2-},\eta /SO_{4}^{2-},Fe/As,$$

which has a simple accuracy, again in full training mode, of 0.67. Again, the accuracies obtained in test mode by our model are sensibly higher, and the recall values, which are, respectively, 0.53, 0.48, 0.70, and 0.25, follow a similar pattern.

## 6 Conclusions

In this paper we have considered the results of the geochemical analysis of groundwater samples from 57 water wells located in the province of Ferrara, all belonging to the same geological group, called group *A*. The hydro-stratigraphic units of interest, which form this group, are in turn formed from one or more depositional sequences characterized by cyclic alternations of fine and coarse deposits. Within each sequence, there are deposits composed by different lithologies, corresponding to various systems and depositional environments, and at the base of each sequence is a very constant level to low permeability that acts as acquiclude, identified between the different units. We considered the problem of identifying the geochemical fingerprint of each aquifer of this group, so that those wells that extract water from the same group but from an unknown aquifer can be safely assigned one, without making decisions based on the depth of the well itself. We proved that our method, based on an artificial intelligence technique which we called feature selection for centroid identification, returns fingerprints with a high level of accuracy, sensibly higher than the one that can be obtained with purely statistical algorithms. Also, as expected, fingerprints that have been obtained using simple characteristics are less precise than those obtained using ratios among elements, as the latter can better identify the geochemical signature of an aquifer, being related to the geochemical signature of the aquifer rocks.

## Declarations

### Author contribution statement

**A. Di Roma:** Conceived and designed the experiments; Contributed reagents, materials, analysis tools or data. **E. Lucena-Sánchez:** Performed the experiments; Analyzed and interpreted the data. **G. Sciavicco:** Conceived and designed the experiments; Contributed reagents, materials, analysis tools or data; Wrote the paper. **C. Vaccaro:** Contributed reagents, materials, analysis tools or data; Wrote the paper.

### Funding statement

The authors acknowledge the partial support from the following projects: Articial Intelligence for Improving the Exploitation of Water and Food Resources, founded by the University of Ferrara under the FIR program, and New Mathematical and Computer Science Methods for Water and Food Resources Exploitation Optimization, founded by the Emilia-Romagna region, under the POR-FSE program.

### Data availability statement

Data will be made available on request.

### Declaration of interests statement

The authors declare no conflict of interest.

### Additional information

No additional information is available for this paper.
